# Therapeutic Development in Charcot Marie Tooth Type 1 Disease

**DOI:** 10.3390/ijms22136755

**Published:** 2021-06-23

**Authors:** Pierre Miniou, Michel Fontes

**Affiliations:** 1 InFlectis BioScience SAS, 21 Rue La Noue Bras de Fer, 44200 Nantes, France; pierreminiou@inflectisbioscience.com; 2 Centre de recherche en CardioVasculaire et Nutrition, Aix-Marseille Université, INRA 1260—INSERM 1263, 13005 Marseille, France; 3 Repositioning SAS, 8 Rue Napoleon, 20210 Calenzana, France

**Keywords:** CMT subtype, animal models, therapies, clinical trials

## Abstract

Charcot–Marie–Tooth disease (CMT) is the most frequent hereditary peripheral neuropathies. It is subdivided in two main groups, demyelinating (CMT1) and axonal (CMT2). CMT1 forms are the most frequent. The goal of this review is to present published data on 1—cellular and animal models having opened new potential therapeutic approaches. 2—exploration of these tracks, including clinical trials. The first conclusion is the great increase of publications on CMT1 subtypes since 2000. We discussed two points that should be considered in the therapeutic development toward a regulatory-approved therapy to be proposed to patients. The first point concerns long term safety if treatments will be a long-term process. The second point relates to the evaluation of treatment efficiency. Degradation of CMT clinical phenotype is not linear and progressive.

## 1. Introduction

Among hereditary peripheral neuropathies, the most frequent is Charcot–Marie–Tooth disease (CMT). CMT constitutes a clinically and genetically heterogeneous group of hereditary motor and sensory peripheral neuropathies. On the basis of electrophysiologic properties and histopathology, CMT has been divided into primary peripheral demyelinating (type 1) and primary peripheral axonal (type 2) neuropathies. The demyelinating neuropathies classified as CMT type 1 are characterized by severely reduced motor nerve conduction velocities (NCV) (less than 38 m/s) and segmental demyelination and remyelination with onion bulb formations on nerve. The axonal neuropathies, classified as CMT type 2, are characterized by normal or mildly reduced NCVs and chronic axonal degeneration and regeneration on nerve. Among the CMT1 group, there are X-linked autosomal dominant and autosomal recessive forms of CMT. Distribution of the different mutations has been reported.

The typical presenting symptom is a weakness of the feet and ankles. The initial physical findings are depressed or absent tendon reflexes with a weakness of foot dorsiflexion at the ankle. The typical affected adult has a bilateral foot drop, symmetrical atrophy of muscles below the knee (stork leg appearance), pes cavus, atrophy of intrinsic hand muscles, especially the thenar muscles of the thumb, and absent tendon reflexes in both upper and lower extremities. The life span is not decreased. 

There are only a few epidemiologic studies on the prevalence of CMT disease. The most generally accepted is the study by Skre proposing a prevalence of one affected person in 2500 [1]. Over 80 causative genes of CMT have been identified with different frequencies [2], and many more remain unknown. The natural history of these varffus forms of CMT remains poorly understood, at least in part, because these are rare disorders and individual centers do not follow enough patients to perform natural history studies. Furthermore, validated clinical instruments for measuring disease severity have become available only recently and have not yet been employed in many of the rare CMT subtypes. The Inherited Neuropathies Consortium (INC) is a member of the Rare Diseases Clinical Research Network (RDCRN) and was created in part to perform natural history studies in CMT. Quantifiable clinical data add to the literature in providing the clinical severity of a variety of CMT subtypes and also act as a baseline for a longitudinal natural history study of CMT subtypes, a prerequisite for clinical trials.

From a recent study of the consortium published in 2015, the frequency of different CMT subtypes ranged from 62% of patients with a genetic diagnosis for the most frequent subtype (CMT1A), up to 0.1% for CMT1D [3]. This review will only focus on CMT type 1 representing more than 80% of CMT disease and thus the mainly explored subtype. 

Therapeutic development in CMT1 relies heavily on the use of non-clinical animal models, cell culture models and more recently, patient-derived cell lines. This review will be divided into two chapters. We will describe first the reports on animal and cellular models that have been constructed and that have been recognized as valuable tools by both the research community and regulators. In a second part, we will describe preclinical and clinical trials performed in different CMT1 subtypes.

## 2. Preclinical Models Used in Therapies Development

Construction of pertinent animal models is a prerequisite stage in drug development. Several models of CMT type 1, as well as cellular models have been reported. We will describe the characteristics of models that have been used to develop the below mentioned therapies. Other models will be only briefly cited.

### 2.1. CMT1A

CMT1A is caused by over expression of the gene *PMP22* caused by duplication of a 1.6 Mb segment of chromosome 17 short arm encompassing this gene [4,5]. Therefore, constructions of CMT1A animal models have been targeted on over expression of this gene. In 1996, the group of K. Nave generated a transgenic rat model of CMT1A by inserting a BAC containing the *PMP22* gene in the murine genome [6]. PMP22-transgenic rats develop gait abnormalities caused by a peripheral hypomyelination, Schwann cell hypertrophy (onion bulb formation), and muscle weakness. Reduced nerve conduction velocities closely resemble recordings in human patients with CMT1A. When bred to homozygosity, transgenic animals completely fail to elaborate myelin.

At the same time, using YAC DNA, a French/English collaboration created a mouse model of a CMT1A by the overexpression of PMP22 human gene. The use of YAC DNA for making transgenic mice has the advantage that the gene of interest, in this case the *PMP22* gene, is surrounded by a large amount of DNA. Five lines of transgenic mice carrying increasing copies of the human *PMP22* gene (one to seven) and expressing increasing levels of the transgene were obtained [7]. From histological and electrophysiological observations, it appears that there is a threshold below which expression of PMP22 has virtually no effect; thus, below a ratio of human/mouse mRNA expression of ~0.8, little effect is observed. Between a ratio of 0.8 and 1.5, histological and nerve conduction velocity abnormalities are observed (C61 line). An expression ratio >1.5 leads to a severe neuropathy (C22 line) [7]. Another observation concerns the histology of the different lines; the level of expression does not affect the type of demyelination but influences the severity of involvement. These data demonstrated that the severity of the clinical phenotype is correlated to the level of PMP22 overexpression [8]. Thus, lowering PMP22 overexpression could be an interesting strategy for phenotypic correction.

More recently, Baas et al. described the phenotype of a spontaneous deletion of the C22 line, and named it the C3 line, which is similar to the C61 line [9].

### 2.2. CMTX1

X-linked Charcot–Marie–Tooth disease (CMTX) is an inherited disorder, presenting as peripheral neuropathy that affects males more severely than females [10]. The average age of onset is ~16 years for males and 19 years for females. It presents with slow muscular atrophy and weakness, predominantly affecting distal muscles. Demyelinating and axonal anomalies are present, making it difficult to distinguish from other CMT forms by clinical evaluation alone.

CMTX1 is caused by mutations in the *GJB1* gene located on the proximal long arm of the X chromosome [11]. It encodes connexin 32 (Cx32), a gap junction protein present in myelin of the peripheral nervous system (PNS) and central nervous system (CNS) [12]. Cx32 is a membrane protein located in gap junctions, which forms hexameric hemichannels called connexons. The docking of two connexons across the intercellular gap triggers the formation of a channel that connects the cytoplasm of adjacent cells and allows the exchange of ions, small molecules (<1000 Da) and signaling effectors [13,14,15]. Pathogenic mutations of the protein affect the function of the channel.

The first mouse model presenting with a neuropathy similar to that seen in patients is a null allele produced by Gjb1 invalidation. This line presents a peripheral neuropathy and abnormalities in myelin [16]. This model is useful for studying the pathophysiology of peripheral nerves linked to the absence of Cx32. However, it could not be used to identify drugs that may correct biochemical defects in the protein seen in experimental models and patients (cell trafficking and connexon deficiency). 

Therefore, a mouse expressing a human mutated Cx32 was generated. The mutations G12S and S26L, found in non-related CMTX families that affect trafficking of Cx32 (G12S) or connexon activity (S26L), have been introduced into a human BAC containing the *GJB1* gene to create mouse transgenic lines. Five transgenic lines were generated and have been investigated [17].

Defects in connexon activity and genomic instability was observed cell lines of the five transgenic lines, suggesting that Cx32 has a role in mitosis. These lines showed locomotor impairment, with the severity of at least the S lines being correlated with the number of copies of the transgene inserted into the murine genome [17].

### 2.3. CMT1B

The CMT1B subtype is caused by mutation in the P0 gene, coding for the major protein of myelin and involved in the folding and stability of myelin sheet [18]. Some mutations in P0 cause the severe early onset neuropathies designated as Dejerine–Sottas disease (DSS), others cause a “classical” CMT1B phenotype with normal early milestones but development of disability during the first two decades of life, and some other mutations cause an adult-onset neuropathy with normal nerve conduction velocities, designated as a “CMT2” form of CMT1B.

Many different genetically modified P0 mice have been generated. P0 null mutant mice have been considered as a model of patients with DSS that are homozygous for functional null mutations. The heterozygous P0 null mutant mouse represents a late onset, milder neuropathy, and has been considered as a model for CMT1B patients carrying a loss of function mutation in one allele [19]. The majority of human P0 mutations is heterozygous and causes a more severe phenotype than that of heterozygous P0-deficient mice. It has been suggested that these mutations act through gain of function. The possibility that some mutations may act through gain of function and others through loss of function could explain, at least to some extent, the findings that patients carrying mutations in the P0 gene are either affected by the mild form of CMT1B or more severe forms of the disease.

Wrabetz 2006 [20] produced two transgenic mice by random insertion of a transgene in which either the P0S63C (DSS) or P0S63del (CMT1B) mutations was inserted. Both mutant alleles produce demyelinating neuropathy that mimics the corresponding human disease. However, P0S63C creates a packing defect in the myelin sheath, whereas P0S63del does not arrive to the myelin sheath and is instead retained in the endoplasmic reticulum, where it elicits an unfolded protein response (UPR). 

A P0 R98C “knock-in” mouse model of CMT1B, where a mutation encoding R98C was targeted to the mouse *Mpz* gene, has been also generated by Saporta [21]. Both heterozygous (R98C/+) and homozygous (R98C/R98C) mice develop weakness, abnormal nerve conduction velocities, and morphologically abnormal myelin, with R98C/R98C mice being more severely affected. Interestingly, MpzR98C is retained in the ER of Schwann cells and provokes a transitory, canonical UPR.

More recently, Fratta 2019 [22] developed a knock-in mice harboring P0Q215X mutation also find in patients. P0Q215X acts through dose-dependent gain of abnormal function and is responsible for P0 mislocalization to non-myelin plasma membranes and induces defects in radial sorting of axons by Schwann cells. Unlike many other P0 mutations, P0Q215X does not elicit an UPR. 

Different types of gain of abnormal function produce the diverse neuropathy phenotypes associated with P0, rendering challenging the development of a unique future therapeutic strategy for CMT1B patients. However, it seems that many CMT1B causing mutations activate the UPR [23]. 

### 2.4. CMT1E

CMT1E, which represents a rare subtype of CMT1, is caused by point mutations in the *PMP22* gene [24,25], which is expressed by Schwann cells and found in peripheral myelin. Clinical manifestations range from mild forms reminiscent of hereditary neuropathy with liability to pressure palsy (HNPP, due to PMP22 haploinsufficiency) to very severe forms of dysmyelinating neuropathy. The CMT1E mice bearing the autosomal dominant trembler mutation (Tr) is by far the most used and studied CMT1E animal model. Tr mice carry a spontaneous L16P mutation in Pmp22 also found in humans and display pathological phenotype resembling the severe dysmyelinating CMT1E found in patients with the same mutation. It manifests by impaired motor development, Schwann cell defect characterized by severe hypomyelination and continuing Schwann cell proliferation throughout life, myelinated fibers loss and severely reduced nerve conduction velocity [26]. Affected animals move clumsily and develop tremor and transient seizures at a young age. The phenotype of Tr mutants is severe and closer to Dejerine–Sottas diseases (CMT3) than to CMT1A.

## 3. Therapeutic Development, from Preclinic to Clinical Trials

### 3.1. Therapies Focused on the Primary Cause of CMT1

#### 3.1.1. CMT1A

As described above, CMT1A disease is caused by overexpression of peripheral myelin protein-22 (*PMP22*) gene. Thus, two parties described the molecular dissection of the PMP22 promoter to find potential biological upstream sequences to reduce gene transcription.

To propose a mechanism of regulation of PMP22 expression, Desarnaud transiently transfected rat Schwann cells with reporter constructs in which luciferase expression was controlled by the promoter region of either the PMP22 or the P0 genes [27]. They report that progesterone stimulated the P0 promoter and promoter 1, but not promoter 2, of PMP22. This effect was specific, as estradiol and testosterone only weakly activated promoters. Thus, the activation of promoter activity of two peripheral myelin protein genes by progesterone is Schwann cell-specific. Using a similar approach, Saberan subcloned genomic fragments covering 6kb of the promoter region of PMP22 and cloned them in an expression vector containing the beta-galactosidase as reporter gene and used it in transfection assays [28]. They showed that the 300 bp upstream of the transcription start contain the elements required for Schwann cell specific expression of the reporter gene. This minimal promoter activity appears to be under the control of a silencer element sensitive to cAMP, located between −0.3 kb and −3.5 kb from the start of transcription. Computer analysis of 2 kb of the promoter predicted the presence of transcription factor binding sites, including CREB (which may be involved in the response of PMP22 expression to cAMP stimulation) and steroid receptors. Using constructs with or without the CREB sites, they were able to demonstrate that these sites are involved in silencing of the PMP22 promoter activity. 

As a consequence of the preceding observations, male transgenic rats were randomly assigned into three treatment groups with progesterone, progesterone antagonist (onapristone), and placebo control. Daily administration of progesterone elevated the steady-state levels of *Pmp22* and *Mpz* mRNA in the sciatic nerve, resulting in enhanced Schwann cell pathology and a more progressive clinical neuropathy. In contrast, administration of the selective progesterone receptor antagonist reduced overexpression of *Pmp22* and improved the CMT phenotype, without obvious side effects, in wild type or transgenic rats [29]. Taken together, these data provide proof of principle that the progesterone receptor of myelin-forming Schwann cells is a promising pharmacological target for therapy of CMT1A. Unfortunately, a clinical trial evaluating Ulipristal acetate, a selective progesterone receptor modulator, in CMT1A patients has been prematurely discontinued (Clinical trial N° NCT02600286) on the request of European and French regulatory agency due to serious liver injury in patients treated with ulipristal acetate in another unrelated clinical trial. Despite this discontinuation of Ulipristal acetate, other progesterone antagonists and/or derivatives could be a future path of investigation in CMT1A.

It has been suggested that ascorbic acid (AA; vitamin C) promotes myelination in axon/Schwann cells [30]. C22 mice were treated either with AA or a placebo, and studied physiologically, using a battery of different tests [31]. All AA treated animals either stop their loss in locomotor performances (rotarod tests) or improved after the first month of treatment (beam walking and grip tests). This improvement continued for the duration of the treatment. In conclusion, these results suggest that treatment with high doses of AA led to a correction of the neuropathic phenotype in transgenic mouse model. In addition, it was demonstrated that AA treatment promotes remyelination in CMT mouse. Moreover, it was shown that a high dose of AA downregulates human *PMP22* expression [32]. Further experiments confirmed these results and suggested that this was due to the mediation of cAMP pool, confirming transient transfection experiments using a reporter gene under the control of the human *PMP22* promoter suggesting that AA may act on *PMP22* expression by modulating cyclic AMP pool. It has been previously shown that *PMP22* overexpression acts by a nonlinear mechanism, with a threshold level of 70–80%. Below this threshold, overexpression can be tolerated without induction of the pathological phenotype [31]. Further experiments revealed that AA is a mild inhibitor of adenylate cyclases, acting as a global regulator of cAMP pool [33]. Because AA is a well-characterized molecule and its pharmacodynamics and toxicity have long been studied, several phase II clinical trials have been initiated. Unfortunately, the design of these studies was not to scale, especially regarding the concentration used in clinical trials. This is important as it has been demonstrated that high concentrations of AA are necessary to act on PMP22 overexpression. Only one trial used high concentration, 3 g/day, the French trial. The report of this trial showed that this dose is safe and that long-term concentration (one year) increased (50 mg/L for placebo, 85 for 1 g/day, and 110 for 3 g/day) [34]. The CMTNS score, a mix of a clinical score (CMTES) and NCV, was used. Although a tendency was observed for the 3 g, it did not reach significance. However, using only the clinical score (CMTES) results reached statistical significance, but this has been evaluated post hoc and not included in primary outcomes. This asks the question of evaluation of results of clinical trials using a same and consensus score. 

At the same time, Burns et al. report a pediatric double blinded trial performed on a cohort of children with CMT1A treated either with a placebo or ascorbic acid at a dosage similar to adults. Placebo and treated children were evaluated using regression curves methods. The two curves presented different slopes, but p-value was at the limit of statistical significancy (0.06) [35].

Two further recent publications suggest the interest of AA in CMT1A. One publication demonstrated that mice invalidated for the transporter regulating blood concentration of AA, SVCT1, presented a demyelinating phenotype similar to CMT [36]. A second paper reports the results of a chemical library screening, using an in vitro myelination system. AA. The molecule emerging from the screening and present remyelinating properties was AA [37].

More recently, the polytherapy product (PXT3003) of Pharnext (France), a mix of three different repurposed molecules (sorbitol, naltrexone, and baclofen), was tested on Schwann cells as well as in the rat model of CMT1A [38]. Their ability to lower Pmp22 mRNA in Schwann cells was assessed in a clonal cell line expressing this gene. These tests showed that treatment with PXT3003 lowers PMP22 overexpression although the mechanism was not unraveled. Furthermore, in vivo efficacy of the combination was tested in two models: CMT1A transgenic rats and mice. The treatment improved myelination in the Pmp22 transgenic co-culture and cellular model. In transgenic CMT1A rats improved myelination of small fibers, increased nerve conduction and ameliorated the clinical phenotype. A phase II clinical trial, double blinded vs. placebo was performed using two doses of PXT3003. Results demonstrated the safety and tolerability of the product [39]. Two score were used: CMTNS and ONLS. Low dose treatment did not show an effect. At the contrary, patients treated with high dose present a significative increase in both score (p-value between 0.04 and 0.05). A first Phase 3 study to further evaluate the efficacy and safety of PXT3003 was conducted in Europe, the United States, and Canada from December 2015 to end of 2017. Two dose levels (highest dose used in the Phase 2 and twice that dose) of PXT3003 in comparison to placebo were tested in 323 mild-to-moderate CMT1A patients. Due to an unexpected formulation issue occurring in the high dose, this treatment arm was prematurely stopped in September 2017. Following the formulation issue that occurred during the first Phase 3 study, the FDA and EMA have requested an additional Phase 3 trial to confirm the efficacy and safety of PXT3003. This trial, called PREMIER, will be an international, multicenter, randomized, double-blind, placebo controlled, pivotal Phase 3 trial. One dose level of PXT3003 (high dose of the first Phase 3) will be tested versus placebo in mild-to-moderate CMT1A patients over a 15-month period. Approximately 350 patients will be enrolled at approximately 50 centers worldwide. The first patient has been enrolled in 1Q 2021. 

Recently, curcumin appeared as a potential promising therapy for CMT, but its development is hindered by its unfavorable pharmacokinetics. Recently, cyclodextrin/cellulose nanocrystals of curcumin (Nano-Cur) have been developed to bypass this limitation. Therapeutic potential of Nano-Cur was investigated in vitro in Schwann cells (SCs) and in vivo in the transgenic CMT1A rat model [40]. In vitro, Nano-Cur treatment (0.01 µM for 8 h) reduced reactive oxygen species and improved mitochondrial membrane potential in CMT1A SCs. Moreover, Nano-Cur treatment (0.01 µM for 1 week) increased the expression of myelin basic protein in SC/neuron co-cultures. Preliminary in vivo experiments carried out in WT rats showed that intraperitoneal (i.p.) injection of Nano-Cur treatment containing 0.2 mg/kg of curcumin strongly enhanced the bioavailability of curcumin. Afterwards, in 1-month-old male CMT1A rats, Nano-Cur treatment (0.2 mg/kg/day, i.p. for 8 weeks) significantly improved sensori-motor functions (grip strength, balance performance, and mechanical and thermal sensitivities). Importantly, sensory and motor nerve conduction velocities were improved. Further histological and biochemical analyses indicated that myelin sheath thickness and myelin protein expression (myelin protein zero and PMP22) were increased. The authors suggested that this action is due to anti-oxidant property of curcurmin. In conclusion, Nano-Cur appears a good candidate to be tested in clinical trials.

Several gene silencing therapy approaches are currently developed in CMT1A. As CMT1A is a gene dosage disease, therapies using antisense oligonucleotide (ASO) to lower PMP22 expression mRNA have been proposed. Ionis Pharmaceuticals (Carlsbad, CA, USA) has published, in collaboration with US CMT patient association CMTA, promising results [41] which show that antisense oligonucleotides (ASOs) effectively suppress PMP22 mRNA in affected nerves in two murine CMT1A models. MNCV and CMAP almost reached levels seen in WT animals. In addition to disease-associated gene expression networks that were restored with ASO treatment, they also identified potential disease biomarkers through transcriptomic profiling. Another company, DTx Pharma (San Diego, CA, USA), has also recently announced a PMP22 ASO program. Zhao results support the use of ASOs as a potential treatment for CMT1A and elucidate potential disease and target engagement biomarkers for use in future clinical trials. Potential limitations associated with this approach, are the recurrent injection of ASO in the organism, the ability to deliver ASO to the Schwann cells, and the level of silencing PMP22 mRNA; indeed, it is critical to reach the right PMP22 mRNA level in Schwann cells because PMP22 haploinsufficiency results in Hereditary Neuropathy with Liability to Pressure Palsies (HNPP) and PMP22 duplication leads to CMT1A. 

Finally, a Korean group [42] performed a direct local intraneural delivery of CRISPR/Cas9, designed to target TATA-box of PMP22, in C22 mice before the onset of disease, and showed the downregulation of *PMP22* gene expression and the preservation of both myelin and axons. Interestingly, the same approach was effective in the partial rescue of demyelination even after the onset of disease. 

#### 3.1.2. CMTX1

As reported, mutation in the *Gjb1* gene, coding for a connexin (Cx32), is associated with X-linked Charcot–Marie–Tooth (CMTX1). The group of K. Kleopa reports the construction of a lentiviral vector carrying the *GJB1* gene under the Schwann cell-specific myelin protein zero (*Mpz*) promoter. This construction was delivered into the mouse sciatic nerve by a single injection immediately distal to the sciatic notch. Enhanced green fluorescent protein (EGFP) reporter gene expression was quantified, and Cx32 expression was examined on a Cx32 knockout (KO) background [43]. They report that EGFP was expressed throughout the length of the sciatic nerve in up to 50% of Schwann cells starting 2 weeks after injection and remaining stable for up to 16 weeks. Gene therapy trial by intraneural injection in groups of 2-month-old Cx32 KO mice, before demyelination onset, significantly reduced the ratio of abnormally myelinated fibers (*p* = 0.00148) and secondary inflammation (*p* = 0.0178) at 6 months of age compared to mock-treated animals. It has been concluded that gene delivery using a lentiviral vector leads to efficient gene expression specifically in Schwann cells. Restoration of Cx32 expression ameliorates nerve pathology in a disease model and provides a promising approach for future treatments of CMTX1. However, this group reports that this construction can correct phenotypes in a mouse line but poorly or an absence of correction on two other mutations. In addition, the transition of this strategy from animal models to patients could be difficult [44].

As reported above, transgenic animals expressing a human mutated Gjb1 transgene present polyploidy and abnormal over-duplication of the centrosome and is associated with locomotor deficit. It has been shown that genomic instability and centrosome duplication is linked to CamKII activity [45]. Exploration of transgenic cell lines revealed that these cells exhibit CamKII over-stimulation [46], a phenomenon that has been linked to mitotic instability (polyploidy, nuclear volume, and centrosome over-duplication); this phenotype is reversed by CamKII inhibitors. It has been also demonstrated that connexon activity is partially restored in transgenic cells using CamKII inhibitors. Regarding in vivo phenotype, it has been shown that degradation on the rotarod test in transgenic mice is significantly lowered by treatment with a CamKII inhibitor (KN93). This effect was seen in two lines with different point mutations in GJB1, and stopping the treatment led to degradation of the phenotype. Moreover, the same group demonstrated that cells from CMTX1 patients present the same phenotype than the cells from transgenic mice and are corrected by treatment with KN93 [47] (for review see in [48]).

#### 3.1.3. CMT 1B

The first publication of a potential therapeutic solution for CMT1B was to treat the disorder by oral administration of curcumin [49]. They demonstrate that treatment with curcumin abrogates endoplasmic reticulum retention of mutants of P0. It relates this work to the above paragraph.

In demyelinating CMTs, the alteration of myelin sheath triggers impairment of axonal excitability that is accompanied by alterations in voltage dependent ion currents, leading to a decrease of the peripheral nerve conduction velocity. An ectopic expression of the sensory neuron specific VGSC isoform Nav1.8 has been reported on motor axons of mild and severe CMT1B mice model, respectively, heterozygously [50] or homozygously [51] deficient for P0 protein. The oral treatment of P0^+/−^ mice with Compound 31, a Nav1.8 inhibitor (Abbevie Inc., Lake Bluff, IL, USA), has been shown to improve animal motor performance, which is associated with an improvement of the amplitude of the plantar compound muscle action potential [52]. Recently, Nav1.8 dysfunction has also been reported in a CMT1B patients [53]. Thus, Nav1.8 blocker represents a promising neuroprotective treatment in demyelinating CMTs.

Mutations in the main structural proteins of myelin, PMP22, P0, and Connexin32 (Cx32), represent the vast majority of all CMT cases being, respectively, responsible for CMT1A, CMT1B, and CMTX1. Some mutant proteins display abnormal trafficking and do not reach the cell membrane but accumulate in the Endoplasmic Reticulum/Golgi apparatus and undergo endosomal and proteasomal degradation (for review see in [54]). The basal endoplasmic reticulum quality control systems can be overwhelmed, and the adaptive stress responses may become insufficient to prevent pathogenesis. The excess of unstructured proteins in the ER triggers an adaptive signal transduction pathway, called unfolded protein response (UPR), which in turn potentiates ER quality control activities to reduce the levels of aberrant molecules. UPR activation has been showed to be triggered in the Trembler-J mouse (Tr-J) by the PMP22-L16P mutation found in CMT1E patients and also in several models of CMT1B (see above paragraphs). 

Numerous studies have shown that enhancing ER quality control pathways and the folding/degradation of proteins may provide a feasible and efficient therapeutic strategy in conformational diseases. Genetic and pharmacological treatments boosting the UPR by inhibiting PP1c/PPP1R15A phosphatase complex have been used successfully in preclinical models of CMT1B. The small molecule IFB-088 (also named Sephin1), a PP1c/PPP1R15A phosphatase complex inhibitor developed by InFlectis BioScience (France), has been shown to ameliorate myelination and to largely prevent the motor, morphological, and molecular defects of S63del mice [55]. A phase 1 study with IFB-088 has been completed in healthy volunteers (EudraCT number: 2018-000443-29; ClinicalTrials.gov Identifier: NCT03610334), and the results of the phase 1 repeated dose-escalation study support the safety and good tolerability profile of IFB-088 in human. Thus, InFlectis BioScience is preparing a phase 2 clinical trial in CMT1. A pharmacological treatment boosting the UPR, like IFB-088/sephin1 could have direct implications for different CMT subtypes where protein misfolding, protein mistrafficking, and ER retention have been observed.

### 3.2. Therapies Focused on Downstream Targets

#### 3.2.1. Neuroinflammation

The group of R. Martini identified low-grade inflammation as a substantial disease modifier in the pathogenesis of distinct CMT1 mouse models (for review see in [56]). While in models for CMT1A, macrophages were identified as the only inflammation related disease modulators, disease outcome in models for CMT1B and CMTX1 is influenced by components of both the innate and the adaptive immune system. They investigated the role of antibodies in a demyelinating model for CMT1B and demonstrated that endoneurial tubes of peripheral nerves of P0het myelin mutant mice are decorated with endogenous antibodies. Furthermore, by cross breeding P0het mice with mouse mutants specifically lacking B-lymphocytes and antibodies (JHD^−/−^), they showed a significant amelioration of demyelination and a reduction of macrophages in peripheral nerves of young P0het JHD^−/−^ mice. Passive systemic transfer of antibodies (IgGs) into P0het JHD^−/−^ mice restored antibody decoration and reverted/reduced macrophage elevation and impaired nerve histology, suggesting a role of endogenous antibodies in macrophage-mediated demyelination. 

Previous studies in myelin-mutant mouse models of CMT have demonstrated that low-grade secondary inflammation implicating phagocytosing macrophages amplifies demyelination, Schwann cell dedifferentiation, and perturbation of axons. The cytokine colony stimulating factor-1 (CSF-1) acts as an important regulator of these macrophage-related disease mechanisms, as genetic and pharmacologic approaches to block the CSF-1/CSF-1R signaling result in a significant alleviation of pathological alterations in mutant peripheral nerves. In mouse models of CMT1A and CMTX1, as well as in human biopsies, CSF-1 is predominantly expressed by endoneurial fibroblasts, which are closely associated with macrophages, suggesting local stimulatory mechanisms. These results further corroborate the important role of secondary inflammation in mouse models of CMT1 and might identify specific targets for therapeutic approaches to modulate innate immune reactions.

However, this approach raised the question of safety of long-term administration of anti-inflammatory molecules. We will comment this point in conclusion chapter.

#### 3.2.2. Muscle Weakness

Muscle atrophy and thus weakness are the main consequences of anomalies in peripheral nerves. Follistatin is a secreted protein that promotes muscle growth and function by sequestering these ligands extracellularly. Acceleron constructed a locally acting, follistatin-based fusion protein, ACE-083, and evaluated the potential of this construction as a novel therapeutic agent for focal or asymmetric myopathies. Intramuscular administration of ACE-083 caused localized, dose-dependent hypertrophy of the injected muscle in wild-type mice and mouse models of Charcot–Marie–Tooth disease (CMT) with no evidence of systemic muscle effects or endocrine perturbation. Importantly, ACE-083 also increased the force of isometric contraction in situ by the injected tibialis anterior muscle in wild type mice and disease models and increased ankle dorsiflexion torque in CMT mice [57]. These results suggested that ACE-083 could be a therapeutic agent for patients with CMT. Based on these preclinical results Acceleron initiates a Phase 2 trial in a cohort of CMT patients which showed that ACE-083 is safe and improve patients’ total and contractile muscle volume, while lowering the proportion of fat in the muscle. These positive findings supported the advancement to the second part of the study. The dose selected to be used during the second part of the trial was not disclosed. The second part involved 44 patients, who were randomly assigned to receive either the selected dose of ACE-083 or a placebo, once every three weeks, for a period of six months. The main goal of this second part of the study was to assess changes in muscle volume. Secondary goals included evaluating the proportion of fat in the muscle, muscle strength and function, balance, sensory and motor impairments, quality of life, and safety measures. Results showed that patients treated with ACE-083 had a significant increase in mean total muscle volume, compared with those who received a placebo, meeting the trial’s primary goal. However, this benefit did not result in significant improvements in any of the functional and quality-of-life secondary goals. From these results the company decided to halt the project.

#### 3.2.3. Axonal Damages

The company Sarepta Therapeutics (Cambridge, MA, USA), in collaboration with the Nationwide Children’s Hospital (Columbus, OH, USA), is developing neurotrophin 3 (NT-3), a gene therapy candidate designed to treat CMT, with the lead indication being type 1A [58]. They showed that NT-3 promotes nerve regeneration and sensory improvement in Trembler mouse model and in CMT1A patients. This treatment approach does not target the CMT causative gene(s) but the axonal damage common to all length dependent neuropathies. Thus, this NT-3 gene therapy approach could have direct implications for other CMT subtypes [59].

#### 3.2.4. Lipids Metabolism

In patients with CMT1A, peripheral nerves display aberrant myelination during postnatal development, followed by slowly progressive demyelination and axonal loss during adult life. It has been shown that myelinating Schwann cells in a rat model of CMT1A exhibit a developmental defect that includes reduced transcription of genes required for myelin lipid biosynthesis. Consequently, lipid incorporation into myelin is reduced, leading to an overall distorted stoichiometry of myelin proteins and lipids with ultrastructural changes of the myelin sheath. Substitution of phosphatidylcholine and phosphatidylethanolamine in the diet is sufficient to overcome the myelination deficit of affected Schwann cells in vivo [60]. This treatment rescues the number of myelinated axons in the peripheral nerves of the CMT rats and leads to a marked amelioration of neuropathic symptoms. Lipid supplementation is an easily translatable potential therapeutic approach in CMT1A and possibly other dysmyelinating neuropathies. This is an interesting target that need to be confirmed by clinical trials.

## 4. Conclusions

The first comment is that the number of publications greatly increased since 2000, revealing the growing interest of CMT to the researchers and clinicians. This is good news for patients that are waiting for a curative treatment.

The second comment concerns safety. It is very likely that treatments will be a long-term process. Several preclinical studies, using animal models, demonstrate that treatment should not be stopped, as stopping the treatment results in phenotype degradation. Therefore, it would be interesting to test molecules for which we already have long-term safety data available. As CMT disease is a progressive neurodegenerative disorder that does not affect live span, long-term safety is a crucial problem.

The third point relates to the evaluation of treatment efficiency. Degradation of CMT clinical phenotype is not linear but occurs by steps. This implies that natural history of the disorder was reported. This is actually the case for CMT1A subtype but natural history of other CMT1 subtypes need to be more documented. Studies on this point shall allow to propose a consensus process of evaluation, which is not currently the case, as different studies are using different scoring systems. CMTNS is the score that has been generally used. It includes a clinical score, CMTES, and NCV. However, it has been reported that NCV is not correlated to disease severity and is not an accurate measure to evaluate disease evolution. CMTES alone is probably better. In addition, other endpoints, such as measures of fat and fibers in muscle evaluated by MRI, have been proposed. Using a consensus process of evaluation will make comparison of different trials easier. Finally, consensus scores should be proposed for all CMT1 diseases or by subtypes. Answers to these questions will be useful for evaluation of future clinical trials.
